# Investigation of the Characteristics of Telomere Length in Koreans and Its Association With Chronic Metabolic Disorders: A Comprehensive Study

**DOI:** 10.1111/jcmm.70964

**Published:** 2025-12-11

**Authors:** Seung‐Beom Chae, Si Nae Park, Ji‐Hoon Lee, Jin‐Tae Kim, Min‐Jung Song

**Affiliations:** ^1^ Department of BT Research Institute U2Bio Co. Ltd. Seoul Korea

**Keywords:** ageing, diabetes, dyslipidaemia, qPCR, T/S ratio, telomere

## Abstract

Telomeres are repetitive DNA sequences located at the ends of eukaryotic chromosomes, forming protective caps that prevent chromosomal degradation and inappropriate repair during cell division. Telomere shortening has been linked to ageing and various age‐related diseases. In this study, we validated the reference range for telomere length established through the analysis of 1011 Korean individuals to confirm its robustness. Telomere length showed a declining trend with increasing age, with no statistically significant differences observed between sexes. Diabetic and dyslipidaemic groups had shorter telomeres than the control group. These findings suggest that telomere length may be a biomarker of biological ageing and could be linked to metabolic conditions. Future research could build upon these observations to explore telomere dynamics in broader populations and investigate their clinical relevance across diverse health outcomes. Furthermore, longitudinal studies are needed to determine whether telomere shortening is a cause or consequence of these metabolic conditions. Additionally, incorporating advanced methodologies, such as next‐generation sequencing, could enhance our understanding of telomere biology and improve the accuracy of telomere‐based measurements in both research and clinical settings.

**Abbreviations**
CBC: complete blood count
Cq: quantification cycle
Ct: cycle threshold
HbA1c: haemoglobin A1c
HDL: high‐density lipoprotein
IRB: Institutional Review Board
LDL: low‐density lipoprotein
NGS: next‐generation sequencing
PBMCs: peripheral blood mononuclear cells
qPCR: quantitative polymerase chain reaction
T/S ratio: telomere/single‐copy‐gene ratio
TI: telomere index
TRF: telomere restriction fragment

## 1 Introduction

Telomeres are repetitive DNA sequences (*TTAGGG* in humans) located at the ends of eukaryotic chromosomes, forming protective caps that prevent chromosomal degradation and inappropriate repair during cell division [1]. Alongside associated proteins, telomeres maintain chromosomal integrity, support nuclear architecture and ensure complete replication of chromosome termini [2]. DNA polymerase requires an RNA primer with a 3′ hydroxyl group to initiate DNA replication. However, as replication progresses and DNA polymerase advances along the template strand, the primer is removed, leading to gaps at the chromosome ends. Consequently, the newly synthesised DNA strand has a reduced length compared with the original template strand. This attrition eventually leads to replicative senescence, a state in which cells lose their ability to divide [3]. Epel et al. [4] emphasised the importance of telomeres as markers of cellular ageing, linking their dynamics to broader implications for tissue and organismal health.

Telomere shortening has been linked to ageing and various age‐related diseases. Ageing involves the gradual decline of physiological and metabolic functions, and telomeres serve as biomarkers of biological ageing, reflecting cumulative genetic and environmental stresses [5]. Moreover, telomere attrition is the most evident in replicating somatic cells, such as leukocytes, where telomere length inversely correlates with chronological age [6]. Genetic predispositions and environmental factors further modulate telomere length, contributing to significant interindividual variability from birth [7]. Mutations in telomerase, the enzyme responsible for telomere maintenance, have been associated with disorders such as aplastic anaemia [8, 9]. Furthermore, critically short telomeres can compromise genomic stability, exacerbating disease processes, including cardiovascular disorders and cancer [10]. These close associations of telomeres with ageing and disease processes highlight their biological significance.

Various methodologies have been developed over time to measure telomere length, each with unique advantages and limitations (Table 1) [11, 12, 13, 14, 15, 16, 17, 18, 19, 20, 21, 22, 23, 24, 25, 26, 27, 28]. Among these, the telomere restriction fragment (TRF) assay, considered the ‘gold standard’, is used to determine mean telomere length by analysing the distribution of telomeric DNA fragments generated through digestion with restriction enzymes that do not cleave within telomeric sequences [11]. TRF is also widely used to validate new methodologies and optimise their applications across various species and experimental contexts [29, 30, 31, 32]. Quantitative polymerase chain reaction (qPCR) is another widely utilised method for measuring telomere length because of its cost‐effectiveness, low DNA input requirement, high‐throughput capability and broad applicability across various research designs [33]. By calculating the telomere/single‐copy‐gene ratio (T/S ratio), which compares telomeric repeats (T) to a single‐copy reference gene (S), qPCR can be used to estimate telomere length based on quantification cycle (Cq) values, normalising DNA input variations through a stable reference gene [17, 18]. These advantages, combined with its simplicity and adaptability, make qPCR an ideal tool for diverse research applications. Recent advances in long‐read sequencing technologies, such as PacBio HiFi and Oxford Nanopore Technologies [34], have transformed genomic research by enabling detailed analysis of complex regions such as telomeres. With these technologies, telomere length and telomeric variant sequences can be precisely measured and detected, respectively, providing valuable insights into genome stability, ageing and disease mechanisms.

**TABLE 1:** Comparison of techniques for telomere length measurement.

Method	Advantages	Disadvantages	Ref.
TRF (telomere restriction fragment)	Established method	Labour‐intensive	[11, 12]
	Provides average telomere length	Requires large amounts of high‐quality DNA	
	Enables cross‐study comparisons		
FISH (fluorescence in situ hybridisation)	Visualises telomere length in situ within a spatial context	Restricted to fixed cells	[13, 14]
	Quantitative with high resolution	Semi‐quantitative	
		Less dynamic than Flow‐FISH	
Flow‐FISH	High‐throughput analysis for telomere lengths across cell subsets	Cannot provide single‐cell resolution with limited dynamic range	[15, 16]
qPCR (quantitative PCR—T/S ratio)	Cost‐effective and rapid for large‐scale studies	Provides average telomere lengths that are less precise than TRF	[17, 18, 19]
	Requires small DNA amounts		
STELA (single‐telomere length analysis)	Precise measurement of short telomeres	Limited to a few telomeres	[20, 21]
	Suitable for detecting critical telomere attrition	Requires high‐quality DNA	
NGS (next‐generation sequencing)	Comprehensive and sequence‐level resolution	Expensive and computationally intensive	[22, 23]
	Capable of analysing complex telomeric regions		
TeSLA (telomere shortest length assay)	Highly accurate for detecting very short telomeres	Requires advanced equipment and expertise for analysis	[24]
Nanopore sequencing	Allows real‐time, long‐read sequencing compatible with diverse sample types	Higher error rates for repetitive sequences like telomeres	[25, 26]
	Compatible with diverse samples	Computational complexity	
HiFi long read sequencing	Highly accurate for long‐read sequences. Ideal for genome assembly and structural variation analysis	Expensive and requires advanced data analysis tools	[27, 28]
	Enables complete telomere‐to‐telomere assembly	Limited accessibility in resource‐constrained settings	

In this study, we examined the telomere length dynamics in a Korean cohort of 1011 individuals aged 10–90 years. Additionally, we investigated age‐related trends and the association of telomere length with common chronic metabolic disorders in Koreans [35], such as diabetes and dyslipidaemia [35], to explore the potential causal relationships. Given its efficiency and suitability for large‐scale population studies, qPCR was employed to ensure precise telomere length measurement. We aimed to provide an in‐depth understanding of telomere biology in ageing and disease by examining the trends in telomere length among Koreans.

## 2 Materials and Methods

### 2.1 Sample Processing

#### 2.1.1 Sample Collection

Blood samples were collected to analyse telomere length trends in Koreans, investigate their relationship with diseases and validate the data through comparative analysis. In the first collection, 1011 anonymised residual blood samples were obtained from patients who underwent a complete blood count (CBC) ordered by medical professionals or from individuals undergoing health check‐ups between 2018 and 2019. Samples were randomly selected based on the following criteria: normal CBC results [36], storage at 2°C–8°C for < 3 days after collection to ensure sample stability and a minimum blood volume of 2 mL. Samples were excluded if they failed to meet these criteria or showed signs of heamolysis, lipaemia or improper storage (e.g., storage temperature outside 2°C–8°C or storage duration exceeding 3 days). In the second collection, 140 anonymised residual blood samples were obtained from individuals aged 40–60 years who had completed glucose and low‐density lipoprotein (LDL) cholesterol tests between May 2024 and November 2024. These samples were also anonymised and unlinked by any personal identifiers. Among these, 80 samples from the healthy group were used to validate the telomere length classification derived from the first dataset. The remaining 60 samples (30 diabetes, 30 dyslipidaemia) were categorised based on clinical criteria:

1. Control group (*n* = 80): Fasting glucose 60–99 mg/dL, LDL cholesterol ≤ 130 mg/dL and haemoglobin A1c (HbA1c) < 6.5%;
2. Diabetic group (*n* = 30): Fasting glucose ≥ 126 mg/dL and HbA1c ≥ 6.5% [37];
3. Dyslipidaemic group (*n* = 30): LDL cholesterol ≥ 160 mg/dL and high‐density lipoprotein (HDL) cholesterol ≤ 40 mg/dL [38].

For the disease‐based comparison, a subset of 30 samples from the validation (healthy individuals) dataset was selected as a control group for comparison against the diabetic and dyslipidaemic groups.

In this study, we utilised residual blood samples collected in two phases independently, anonymised and unlinked from any personal identifiers. Therefore, the requirement for informed consent was waived. The first and second phases of blood sample collection were approved by the Institutional Review Board (IRB) of U2 Medical Foundation (IRB_2017022_MU01 and IRB_2024026_MU01, respectively).

#### 2.1.2 Sample Preparation

Peripheral Blood Mononuclear Cells (PBMCs) were isolated using Lymphoprep (STEMCELL, Vancouver, BC, Canada) following the manufacturer's protocol. Whole blood samples were processed immediately or stored at 4°C for up to 24 h before PBMC isolation. Isolated PBMCs were immediately used for genomic DNA extraction, performed using the QIAsymphony DSP DNA Mini Kit (Qiagen, Hilden, Germany) according to the manufacturer's instructions. The quality and concentration of the extracted DNA were assessed using a NanoDrop spectrophotometer (Thermo Fisher Scientific, Waltham, MA, USA). Acceptable samples had DNA concentrations at ≥ 50 ng/μL and A260/280 ratios between 1.7 and 2.0. Extracted DNA was initially stored at −20°C for routine use and short‐term storage and at −80°C in sealed containers for long‐term preservation and stability.

### 2.2 Telomere Analysis

#### 2.2.1 Quantitative Polymerase Chain Reaction Setup

Telomere and β‐globin gene primers were synthesised using sequences from a previously published study [18]. The sequences of the telomere primers were as follows:

- Forward: 5′‐ACA CTA AGG TTT GGG TTT GGG TTT GGG TTT GGG TTA GTG T‐3′ (40 bp);
- Reverse: 5′‐TGT TAG GTA TCC CTA TCC CTA TCC CTA TCC CTA TCC CTA CAC‐3′ (42 bp).

For the β‐globin gene, the primer sequences were as follows:

- Forward: 5′‐CGG CGG CGG CGG CGG GCT GGG CGG CTT CAT CCA CGT TCA CCT TG‐3′ (47 bp);
- Reverse: 5′‐GCC CGC GCC GCC GCC GTC CCG CCG GAG GAG AAG TCT GCC GTT‐3′ (45 bp).

The qPCR test was conducted using a 20 μL total volume, which included 12.5 μL of PowerUp SYBR Green PCR Master mix (Thermo Fisher Scientific, Waltham, MA, USA), 1 μL each of telomere forward and reverse primers (final concentration 0.9 μM), 1 μL each of β‐globin forward and reverse primers (final concentration 0.5 μM), 3.5 μL of distilled water and 5 μL of template or standard DNA. The reaction mixture was thoroughly mixed before being subjected to thermal cycling.

The qPCR was performed under the following conditions:

1. Initial denaturation at 95°C for 15 min to ensure complete DNA denaturation;
2. Two cycles of denaturation at 94°C for 15 s and primer annealing at 49°C for 15 s;
3. The main amplification step consists of 34 cycles, each including denaturation at 94°C for 15 s, annealing at 62°C for 10 s, extension at 74°C for 15 s, annealing at 84°C for 10 s and denaturation at 88°C for 15 s.

All thermal cycling steps were conducted using a CFX96 Real‐Time PCR Detection System (Bio‐Rad, Hercules, CA, USA). Additionally, a melt curve analysis was performed from 6°C to 95°C (0.5°C increment every 5 s) to confirm the specificity of the amplified products. The reaction was then held at 4°C indefinitely for storage. Each sample was analysed in duplicate, and the average Ct value was used for further calculation. Positive and negative control DNA samples were included in each run to verify amplification reliability and detect potential contamination.

#### 2.2.2 Telomere Length Measurement

Telomere length was measured using the CCRF‐CEM cell line as a standard for generating the standard curve for quantitative analysis. A five‐point serial dilution (beginning at 20 ng/μL with a 1:2 dilution ratio) was used, and the cycle threshold (*C*_t_) values were confirmed to maintain linearity with an *R* ^2^ ≥ 0.9. T/S ratio was determined based on the standard curve, as outlined in the qPCR method described by Cawthon [17]. The relative T/S ratio was calculated using the formula:

(1) $$\;\left[2^{\left(\Delta Ct\;\left[\text{CCRF}-\mathrm{CEM}\right]-\Delta Ct\;\left[\text{Sample}\right]\right)}\right]=2^{\Delta \Delta Ct}.$$

Δ*C*_t_ was calculated by subtracting the Telomere's *C*_t_ value from β‐globin's *C*_t_ value. Furthermore, telomere index (TI) was determined using the formula:

(2) $$\;\mathrm{TI}=\left(\text{Individual}\;T/S\;\text{ratio}\right)/\left(\mathrm{Age}-\text{specific mean}\;T/S\;\text{ratio}\right).$$

Telomere length was categorised into five arbitrary groups based on the TI values (Table 2).

**TABLE 2:** Telomere index category.

TI category	Range
Very short	0 ≤ TI < 0.7
Short	0.7 ≤ TI < 0.9
Normal	0.9 ≤ TI < 1.1
Long	1.1 ≤ TI < 1.3
Very long	1.3 ≤ TI

Abbreviation: TI, telomere index.

### 2.3 Data Framework Analysis

#### 2.3.1 Demographic Analysis of Korean Subjects

To investigate the relationship between telomere length and demographic factors, we categorised 1011 Korean individuals (aged 10–90 years) into 10‐year age groups. Telomere length was measured using qPCR, and the T/S ratio was analysed. Pearson correlation analysis was applied to assess the relationship between the T/S ratio and age, while independent t‐tests were used to compare telomere length differences between males and females.

#### 2.3.2 Validation of the Established Reference Range

Furthermore, the telomere lengths of an additional 80 healthy Korean individuals from a second collection group (*n* = 140) were analysed to verify the reliability of the telomere length reference range established from the primary dataset (*n* = 1011). Since the guidelines of the Clinical and Laboratory Standards Institute (CLSI EP28‐A3c) recommend a minimum of 20 individuals for initial evaluation, we expanded the number of validation samples to 80 to enhance statistical robustness. The mean and standard deviation (SD) of the T/S ratio values were calculated to assess variability and compare them against the established reference range from the primary dataset.

#### 2.3.3 Correlation With Disease

Participants were classified into three groups (control, diabetic and dyslipidaemic) based on clinical criteria to evaluate the association between telomere length and metabolic disorders. The T/S ratio was measured using qPCR, and the TI was calculated to normalise individual telomere lengths relative to age‐specific references. Chi‐square and Fisher's exact tests were used to evaluate categorical differences in telomere length distributions among the disease groups.

### 2.4 Statistical Analysis

All statistical analyses were conducted using Jamovi (version 2.2) and R (version 4.0). The chi‐square tests were performed using Jamovi, while Pearson correlation analysis, regression analysis, box plots and other visualisations were conducted using R. A *p*‐value < 0.05 was considered statistically significant.

## 3 Results

### 3.1 Telomere/Single‐Copy‐Gene Ratio Across Age Groups and Sex

**FIGURE 1:**
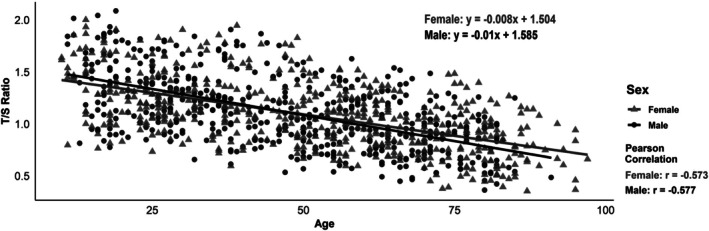
Telomere/single‐copy‐gene ratio (T/S ratio) correlation with age and sex in Korean individuals. A moderate inverse correlation was observed between age and T/S ratio in both sexes (female: *r* = −0.573; male: *r* = −0.577), indicating an age‐related decline in telomere length.

**FIGURE 2:**
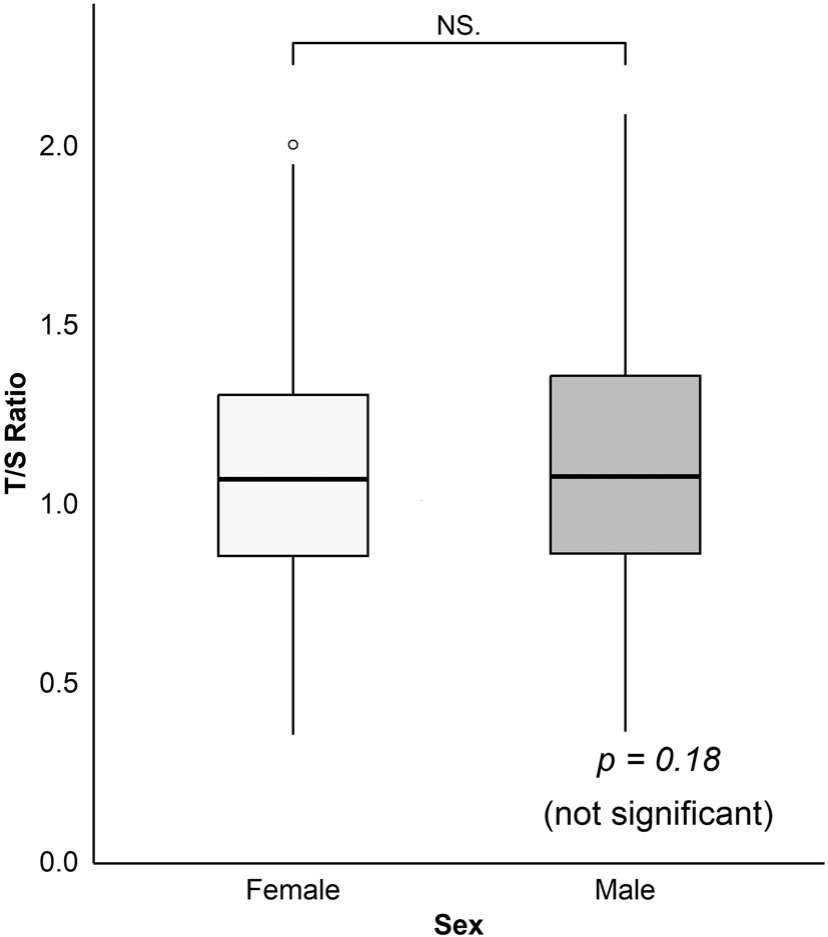
Comparison of telomere length (T/S ratio) between sexes in Korean individuals. No statistically significant difference in T/S ratio was observed between females and males (*p* = 0.18, independent *t*‐test), suggesting comparable telomere lengths across sexes. Raw *p*‐value: 0.1775.

**FIGURE 3:**
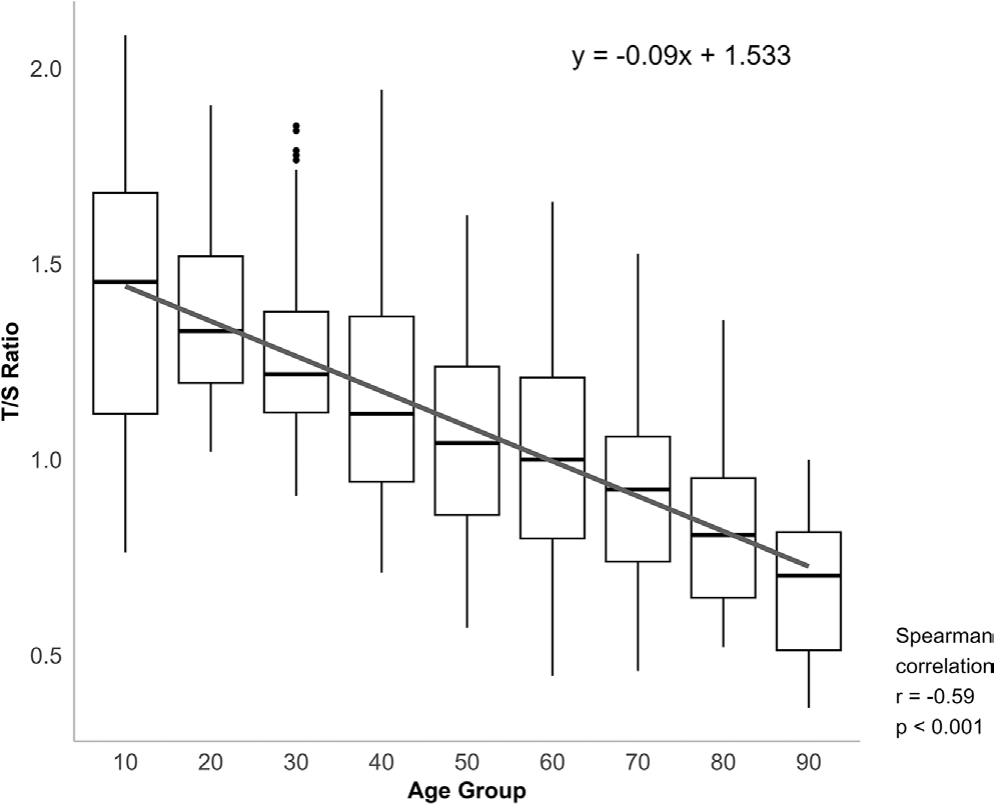
Age‐related decline in T/S ratio across 10‐year age groups in Korean individuals. A significant negative correlation was observed between age group and T/S ratio (Spearman *r* = −0.59, *p* < 0.001), indicating progressive telomere shortening with increasing age. Raw *p*‐value: 3.90 × 10^−82^.

A moderate negative correlation was observed between age and T/S ratio in both males and females (Figure 1). The Pearson correlation coefficients were *r* = −0.573 (*p* < 0.001) and −0.577 (*p* < 0.001) for females and males, respectively, indicating a consistent decline in the T/S ratio with increasing age. Linear regression analysis showed a similar trend in both sexes, with a slightly steeper slope observed in males. However, no statistically significant difference was observed in the T/S ratio between the sexes (*p* = 0.18, Figure 2). Age group analysis (Figure 3) revealed a progressive decline in the T/S ratio with increasing age, which was supported by a statistically significant Spearman correlation (*r* = −0.564, *p* < 0.001). Specifically, the 10‐year‐old group exhibited the highest T/S ratios, with a mean value close to 1.5. Additionally, as age increased, the range of T/S ratio values within each age group gradually narrowed, indicating a reduced inter‐individual variability with ageing.

### 3.2 Validation of Telomere/Single‐Copy‐Gene Ratio Reference Range

We analysed and compared the T/S ratio of 80 healthy individuals (second collection group) against the established range to validate the robustness of the reference range derived from the original 1011 sample dataset (Table 3). Among these 80 healthy individuals, two individuals (2.5%) had T/S ratio values outside the reference range, while 78 (97.5%) were within the range. This result meets the CLSI EP28‐A3c criteria, which require that no more than 10% of values fall outside the reference range for validation studies.

**TABLE 3:** Validation of telomere length reference range with additional samples.

Age group	*N*	T/S ratio			Reference range	Out of range (*N*)
		Min	Max	Mean (SD)		
40s	12	0.87	1.21	1.04 (0.13)	0.87–1.49	1
50s	29	0.76	1.16	0.93 (0.09)	0.78–1.31	1
60s	39	0.71	1.13	0.92 (0.10)	0.74–1.28	0
Total	80					2/80 (2.5%)

*Note:* The reference range was defined as the mean T/S Ratio for each age group, with a margin of variability based on the standard deviation.

### 3.3 Correlation Between Telomere Length and Disease Groups

**FIGURE 4:**
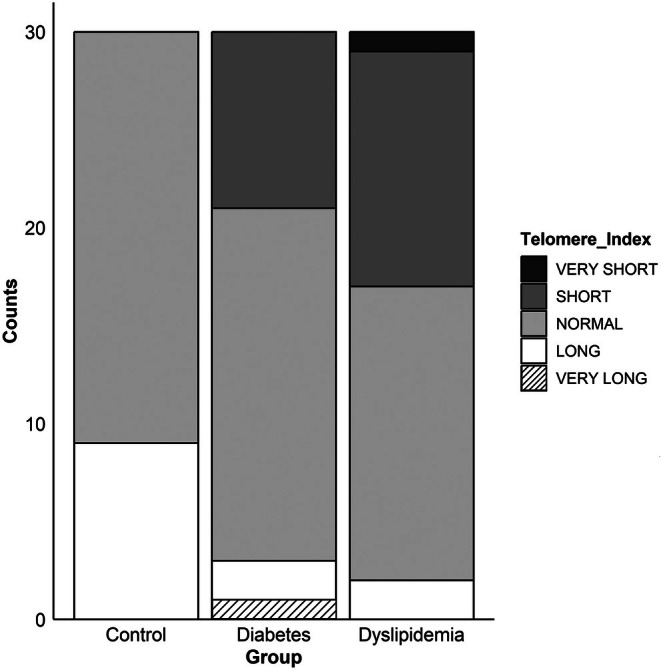
Telomere index classification across control, diabetic and dyslipidaemic groups. Individuals with diabetes or dyslipidaemia demonstrated a marked shift toward shorter telomere index categories, suggesting a potential association between telomere shortening and metabolic disease status.

The comparison of telomere length distributions across control, diabetic and dyslipidaemic groups revealed significant differences (Table 4, Figure 4). In the control group (*n* = 30), most participants fell into the ‘normal’ and ‘long’ TI categories, indicating a healthier telomere profile. Conversely, participants in the diabetic and dyslipidaemic groups showed a marked shift toward the ‘short’ TI category. The distribution of individuals with ‘very short’ telomeres was notably higher in these disease groups than in the control group. Statistical analysis using the chi‐square test demonstrated a significant difference in TI distributions among the three groups (*p* < 0.001). Additionally, Fisher's exact test was used to account for low frequencies in some categories, further confirming the significance (*p* < 0.001). These findings suggest that telomere length varies distinctly between healthy individuals and those with metabolic conditions.

**TABLE 4:** Telomere index comparison and statistical tests in control and disease groups.

Group	Telomere index					
	Very short	Short	Normal	Long	Very long	Total
Control	0	0	21	9	0	30
Diabetes	0	11	16	2	1	30
Dyslipidaemia	1	12	15	2	0	30
Total	1	23	52	13	1	90

	Value	df	*p*	Raw *p*
*χ* ^2^ and Fisher's exact tests (control and diabetic)				
*χ* ^2^	17.1	3	< 0.001	0.00066
Fisher's exact test			< 0.001	0.00035
*N*	60			
*χ* ^2^ and Fisher's exact tests (control and dyslipidaemic)				
*χ* ^2^	18.5	3	< 0.001	0.00006
Fisher's exact test			< 0.001	0.00001
*N*	60			

## 4 Discussion

A consistent declining trend was observed in telomere length across all age groups in this study, reinforcing its role as a robust biomarker of cellular ageing. This consistent decrease with age aligns with previous findings that telomere shortening is a key marker of biological ageing [8]. Our findings in this study showed no significant differences in telomere length between males and females (*p* = 0.18), suggesting that sex may not be a primary factor in telomere length within this cohort. While some studies report that women generally have longer telomeres than men [39, 40], others, such as Okuda et al. [7], suggest no significant sex differences at birth. Interestingly, a meta‐analysis by Gardner et al. [41] found that sex‐based difference in telomere length appears only when measured using Southern blot, whereas methods like real‐time PCR or Flow‐Fluorescence In Situ Hybridization (Flow‐FISH) do not show a significant variation. Since qPCR was utilised in this study, the findings align with previous studies, suggesting that methodological differences rather than biological factors may account for discrepancies in sex‐based telomere length variation.

Studies have shown that telomere length distribution varies across ethnic groups. For instance, Vyas et al. [42] demonstrated that racial and ethnic backgrounds can significantly influence the relationship between lifestyle factors and telomere length, highlighting the need for population‐specific approaches when interpreting telomere data. African Americans tend to exhibit longer telomeres than Caucasians [43, 44]. Given these racial differences, the Korean data presented in this study provide a valuable reference for evaluating telomere length within this population. Unlike previous studies that predominantly focus on Western populations, this dataset provides one of the first large‐scale assessments of telomere length in Koreans, ensuring a more accurate reference framework for future epidemiological and clinical applications.

The validation process confirmed that 97.5% of the 80 healthy individuals assessed fell within the reference range established from the 1011‐sample dataset, meeting the CLSI EP28‐A3c criteria, confirming the reference range. However, the observed 2.5% deviation underscores the inherent biological variability among individuals, which may be influenced by genetic predisposition, environmental factors or measurement inconsistencies. These findings reinforce the robustness of the reference range in real‐world applications.

When comparing telomere index distributions, the control group had a higher proportion of individuals in the ‘normal’ and ‘long’ categories, whereas the diabetic and dyslipidaemic groups showed a noticeable shift toward the ‘short’ and ‘very short’ categories. This distribution suggests that shorter telomeres may be associated with these metabolic conditions, potentially reflecting increased oxidative stress or chronic inflammation. These findings highlight the potential utility of telomere length as a biomarker for disease stratification. Shorter telomeres could serve as indicators of existing metabolic disorders and as predictive markers for individuals at risk of developing these conditions [45, 46]. Additionally, the distinct telomere length distributions observed in the disease groups emphasise the importance of monitoring telomere dynamics in clinical settings. However, the limited sample size for each group (*n* = 30) reduces the generalisability of these results.

Despite its strengths, this study has some limitations. Firstly, the small sample size in the diabetic and dyslipidaemic groups (*n* = 30 per group) limits the statistical power of subgroup analyses and may increase the risk of Type II errors, where true differences between groups may not be detected. Future studies should include larger cohorts and consider a wider range of disease conditions − including cancer, cardiovascular and neurodegenerative diseases, as well as other metabolic disorders such as obesity and hypertension, which are both linked to telomere shortening − to enhance the clinical relevance and broaden the understanding of telomere dynamics in metabolic health. Secondly, this study utilised anonymised, residual blood samples without linked information on lifestyle or socioeconomic factors (e.g., smoking, BMI, stress, income), precluding multivariable adjustment for potential confounders. This is an inherent limitation of retrospective studies using de‐identified samples and highlights the need for prospective designs with detailed metadata collection. Finally, only qPCR was used to measure telomere length. Although efficient, it does not provide absolute values. Incorporating TRF analysis and next‐generation sequencing would improve accuracy and enable both absolute and relative telomere length assessments, enhancing cross‐study comparability and understanding of telomere biology.

## 5 Conclusions

In this study, we established a validated reference range for telomere length in a Korean population and confirmed its robustness across age groups. Telomere length consistently declined with age, reinforcing its role as a biomarker of biological ageing. Additionally, shorter telomeres were significantly associated with diabetes and dyslipidaemia, suggesting that telomere length may reflect metabolic health status. These findings provide a population‐specific foundation for future research into telomere‐related health outcomes in Koreans.

## Author Contributions

**Seung‐Beom Chae:** conceptualization (equal), data curation (equal), formal analysis (lead), investigation (equal), methodology (equal), software (equal), validation (equal), visualization (equal), writing – original draft (equal), writing – review and editing (equal). **Si Nae Park:** conceptualization (equal), data curation (equal), investigation (equal), methodology (equal), software (equal), validation (equal), visualization (equal), writing – review and editing (equal). **Ji‐Hoon Lee:** investigation (equal). **Jin‐Tae Kim:** funding acquisition (lead), resources (lead), writing – review and editing (equal). **Min‐Jung Song:** conceptualization (equal), methodology (equal), project administration (lead), software (equal), supervision (lead), validation (equal), visualization (equal), writing – original draft (equal), writing – review and editing (equal).

## Funding

This work was supported by U2Bio CO. LTD.

## Ethics Statement

This study was conducted in accordance with the Declaration of Helsinki and utilised residual blood samples collected in two phases, anonymised and unlinked from any personal identifiers. Therefore, the requirement for informed consent was waived. The first and second phases of blood sample collection were approved by the Institutional Review Board (IRB) of U2 Medical Foundation (IRB_2017022_MU01 and IRB_2024026_MU01, respectively).

## Consent

Informed consent was waived due to the use of anonymised residual blood samples without any personal identifiers.

## Conflicts of Interest

The authors declare no conflicts of interest.

## Data Availability

The data presented in this study are available upon request from the corresponding author.
